# Contributing factors to healthcare costs in individuals with autism spectrum disorder: a systematic review

**DOI:** 10.1186/s12913-022-07932-4

**Published:** 2022-05-06

**Authors:** Behzad Karami Matin, Sarah Byford, Shahin Soltani, Ali Kazemi-Karyani, Zahra Atafar, Ehsan Zereshki, Moslem Soofi, Satar Rezaei, Shiva Tolouei Rakhshan, Parvin Jahangiri

**Affiliations:** 1 Research Center for Environmental Determinants of Health (RCEDH), Health Institute, Kermanshah University of Medical Sciences, Kermanshah, Iran; 2 King’s College London, London, UK; 3 Social Development and Health Promotion Research Center, Health Institute, Kermanshah University of Medical Sciences, Kermanshah, Iran; 4 Behavioral Disease Research Center, Kermanshah University of Medical Sciences, Kermanshah, Iran

**Keywords:** Autism, Healthcare costs, Out of pocket payments, Determinants, Systematic review

## Abstract

**Background:**

Individuals with autism spectrum disorder (ASD) are more likely to use healthcare than their counterparts without disabilities, which imposes high medical costs to families and health systems. This study aimed to investigate healthcare costs and its determinants among individuals with ASD.

**Methods:**

In this systematic review, we searched online databases (Web of Science, Medline through PubMed and Scopus) for observational and experimental studies that included data on service use and costs associated with ASD and published between January 2000 and May 2021. Exclusion criteria included non-English language articles, duplicates, abstracts, qualitative studies, gray literature, and non-original papers (e.g., letters to editors, editorials, reviews, etc.).

**Results:**

Our searches yielded 4015 articles screened according to PRISMA guidelines. Of 4015 studies identified, 37 articles from 10 countries were eligible for final inclusion. Therapeutic interventions, outpatient visits and medications constituted the largest proportion of direct medical expenditure on individuals with ASD. Included studies suggest lack of health insurance, having associated morbidities, more severe symptoms, younger age groups and lower socioeconomic status (SES) are associated with higher medical expenditure in individuals with ASD.

**Conclusions:**

This systematic review identified a range of factors, including lower SES and lack of health insurance, which are associated with higher healthcare costs in people with ASD. Our study supports the formulation of policy options to reduce financial risks in families of individuals with ASD in countries which do not have a tax-based or universal health coverage system.

## Introduction

Autism spectrum disorder (ASD) is a neurodevelopmental disorder that affects social abilities, communication skills and behavioral patterns among people with ASD [1]. This disorder occurs in all racial, ethnic and socioeconomic groups and its prevalence has grown rapidly in the last few decades [2]. According to the Centers for Disease Control and Prevention, about 1 in 54 children have been diagnosed with ASD [3]. Some studies have reported the prevalence of ASD to be approximately 1–2% in Asia, Europe and North America [4].

Individuals with ASD require behavioral interventions and specialty health services to improve social interactions, communication skills and daily functioning. Evidence suggests that individuals with ASD are more likely to have higher rates of utilization of both acute care (e.g. hospitalization and emergency department visits) and specialty care (e.g. psychiatric and neurology visits) than their counterparts without ASD [5, 6]. For example, Croen and colleagues found that children with ASD experienced a higher percentage of inpatient (3% vs 1%) and outpatient (5% vs 2%) hospitalizations compared to children without ASDs [7]. In addition, people with ASDs tend to use more psychotherapeutic medications than people without ASDs that impose considerable costs on families and health systems [8, 9].

Studies show a strong relationship between co-occurring conditions and healthcare cost among individuals with ASD. Of these, mental health problems have a major effect on medical care costs. Peacock et al. found that children with ASD and intellectual disability incurred expenditures 2.7 times higher than children with ASD and no co-occurring condition [10]. Similarly, a study by Zerbo et al. indicated that adults with ASD had a significantly higher mean number of visits for mental health (43.3% vs 5.4%) and speech therapy (0.8% vs 0.07%) compared with the general population [11].

The economic burden associated with ASD has been investigated in various studies. Studies have reported higher healthcare costs among individuals with ASD compared to the general population. For example, a study by Lavelle et al. indicated that annual costs of healthcare among children with ASDs were more than four times higher (14,061 USD vs 3020 USD) than those without ASDs [12]. Higher utilization of healthcare among individuals with ASDs may additionally impose higher direct costs on families, particularly in countries where out of pocket payment (OPP) for health services is common, and higher indirect costs, as a result of lost or disrupted employment for individuals and their parents/carers. We should note that healthcare is only one element of costs relevant to people with an ASD; other relevant costs include day care, respite care and education, including specialist education [13].

Some factors, such as age, place of residence, the level of disability, and associated comorbidities, can intensify the economic burden carried by individuals with ASDs. For example, Knapp et al. in the UK, found that lifetime costs are substantially higher for individuals with both ASD and intellectual disability than for those with ASD alone (£1.23 million vs £0.80 million) [13]. Similarly, Horlin et al. in Australia, noted that each additional symptom adds around 1400 USD per annum to the cost of an individual with ASD [14]. Timing of diagnosis also influences cost, with studies demonstrating that delayed diagnosis and access to specialty healthcare can increase associated comorbidities in people with disabilities which consequently leads to higher healthcare costs [15–17].

Overall, the consequences of ASD can potentially affect employment, family relationships, standards of living, social interactions, personal functioning, and quality of life for individuals and their families. In this systematic review, we aimed to identify factors associated with healthcare costs among individuals with ASD. Total costs associated with ASD can vary between individuals and between countries, as a result of a number of different factors. However, identification and quantification of the healthcare, and other, costs associated with ASD can provide a comprehensive picture of the financial impact on families and health systems and can help to identify factors affecting those costs, which may be amenable to change or policy intervention. To ensure appropriate planning of the afore-mentioned services and allocation of resources, it is essential to identify the major contributors to healthcare costs among individuals with ASD. Therefore, the present study aimed to identify determinants of healthcare costs among individuals with ASD.

## Method

### Search strategy

Our study was a systematic review that adheres to the PRISMA guidelines [18]. Three online bibliographic databases were used to search English language articles from January 1, 2000 to May 31, 2021: Web of Science, Medline through PubMed and Scopus. Two main foci were used to search for studies: (1) autism; and (2) cost; we used a combination of keywords to search for studies meeting our inclusion criteria and the search strategy is shown in Table 1. In addition, we searched the references of all included studies to identify additional studies.

**Table 1:** Search strategy in the included databases

Databases	Number of abstracts
**Web of science**	1191
TITLE: (autis*) AND TOPIC: (expen*)	215
TITLE: (autis*) AND TOPIC: (cost*)	500
TITLE: (autis*) AND TOPIC: (econom*)	249
TITLE: (autis*) AND TOPIC: (spen*)	94
TITLE: (autis*) AND TOPIC: (financ*)	133
**Medline through PubMed**	1333
(autis*[Title/Abstract]) AND (expen*[Title/Abstract])	183
(autis*[Title/Abstract]) AND (cost*[Title/Abstract])	617
(autis*[Title/Abstract]) AND (econom*[Title/Abstract])	285
(autis*[Title/Abstract]) AND (spend*[Title/Abstract])	97
(autis*[Title/Abstract]) AND (financ*[Title/Abstract])	151
**Scopus**	1474
TITLE-ABS (autis* AND expen*)	199
TITLE-ABS (autis* AND cost*)	665
TITLE-ABS (autis* AND econom*)	313
TITLE-ABS (autis* AND spend*)	124
TITLE-ABS (autis* AND financ*)	173

We included observational (cross-sectional studies, case-control studies, and cohort studies) and experimental (randomized control trial, quasi experimental) studies. As shown in Table 2, inclusion criteria were defined according to the Patient, Intervention, Comparator, Outcome, Study design (PICOS) framework. Our primary outcomes were factors associated with healthcare costs in individuals with ASD. In health economics, healthcare costs are categorized into direct and indirect costs. Direct costs are defined as those costs that are directly attributable to an illness or disorder, including direct medical costs (the cost of healthcare interventions) and direct non-medical costs (additional costs as a result of accessing healthcare interventions, such as accommodation, meals, and transportation costs) [19]. Indirect costs are costs (or ‘losses’) incurred due to the cessation of or reduction in work productivity as a result of the morbidity associated with an ASD [20]. In this study, total costs were the sum of direct and indirect costs.

**Table 2:** PICOS inclusion criteria

PICOS	Criteria
Population	Individuals with ASD
Intervention	N/A
Comparator	N/A
Outcome	Contributing factors to healthcare costs in individuals with ASD
Study design	Observational and experimental studies

### Inclusion criteria

1. Individuals with ASD
2. Studies reporting healthcare costs for individuals with ASD
3. Observational studies (cross-sectional studies, case-control studies, and cohort studies) or experimental studies (randomized control trial, quasi experimental) Published in English between January 1, 2000 and May 31, 2021
4. Full-text articles

### Exclusion criteria

1. Published before January 1, 2000 and after May 31, 2021
2. Qualitative research, case studies, and literature reviews (e.g. scoping and systematic reviews)
3. Abstracts, editorials, letter to editor, and comments
4. Protocols and method papers
5. Grey literature (e.g. books, conference abstracts, theses, research reports, policy documents)
6. Non-English language studies

### Data analysis

We developed a data extraction form to extract information such as authors, year, country, study design, study sample, costs, study perspective (for example: patient, healthcare sector, payer, societal or institutional perspective [21]), and patient characteristics hypothesized to be determinants of healthcare costs (for example, co-morbidities, severity of illness, age, health insurance status, socioeconomic status) from the included studies. Three main steps of title screening, abstract screening and full-text screening were performed to select the included studies. At the first step of title screening, we searched titles using the keywords outlined in Table 1 and then removed duplicate records. At the abstract screening stage, two authors independently checked the titles and abstracts for relevance (AK and MS), removed those clearly meeting exclusion criteria and retained all potentially relevant abstracts for full-text review. Full-text papers were similarly screened independently by two authors to ensure they met the inclusion criteria and did not meet the exclusion criteria (AK and MS). Data were extracted from all included studies using a pre-specified checklist of key study items. The corresponding author checked the accuracy of the data by comparing and adapting the extracted data with the information contained in the included articles. All disagreements were resolved by consensus after discussion. Where the full-text of papers could not be accessed, corresponding authors were contacted by email to request access. We used EndNote X9.3 to screen the papers and extract data.

Regarding the design of the included studies, we applied JBI Critical Appraisal Checklists for cross sectional studies and cohort studies to assess the quality of the studies included in the review [22]. Quality assessment was carried out by two authors independently (AK and MS).

## Results

**Fig. 1:**
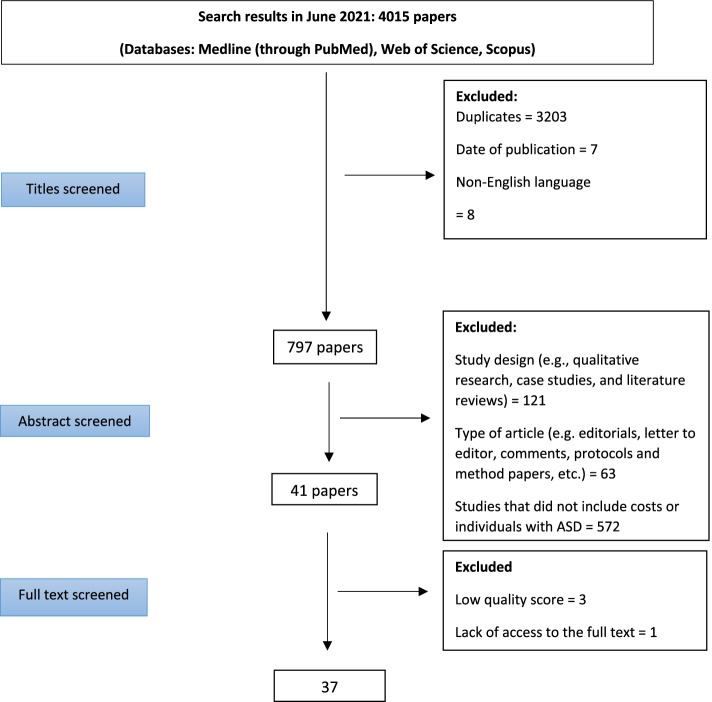
The process of systematic review of the literature

The process of study selection is shown in Fig. 1. In total, our search yielded 4015 papers (including manual searching of reference lists of included papers). In the first step of screening (title screening), 3218 articles were excluded because of language (not English), date of publication outside the dates of interest or because they were duplicates. In the second step of screening (abstract screening), 756 articles were excluded from the study because of study design (qualitative studies or an excluded design), participants (not ASD), type of article (letter to the editor, editorial and review), or for not reporting heath care costs. In the final step (full text screening), a further 4 articles were excluded because of lack of access to the full text or low quality score. Thus 37 articles met criteria for inclusion.

The 37 included studies, which are summarized in Table 3, were carried out in 10 countries: the United states of America (USA; *n* = 21), the United Kingdom (UK; *n* = 6); both USA and UK (*n* = 1),Taiwan (*n* = 2), Ireland (*n* = 1), Germany (*n* = 1), Israel (*n* = 1), China (*n* = 1), Australia (*n* = 1), Oman (*n* = 1), and South Korea (*n* = 1). The age of participants ranged between 1 and 89 years and the majority of studies focused on ASD or ASD with and without intellectual disability.

**Table 3:** Study characteristics

Authous (Year)	Country	Study aim	Data source	Sample size (age)	Study perspective	Type of health services	Costs included in the study			Period of the study
							Average direct costs per person	Average indirect costs per person	Average total costs per person	
Jarbrink and Knapp (2001) [23]	UK	To estimate the economic consequences of autism in the UK	Secondary analysis of data	Autism with additional learning disabilities	Patient Provider	Hospital/inpatient services Medication use Other	OOP: $67,098 Provider spending: $221,027	NR	NR	Lifetime
				High functioning autism			OOP: NR Provider spending: $152,717	$298,256	NR	
Jarbrink (2003) [24]	USA	To show the major cost drivers among children with ASD	Survey (Respondents: parents)	*N* = 17 (4–10 years)	Provider	Early intervention Health services Medication use	Provider spending: $416	NR	NR	Weekly
Liptak et al. (2006) [25]	USA	To compare healthcare expenditures between children with ASD and other children with and without disabilities	Survey (Respondents: parents)	*N* = 100 (< 17 years old)	Patient	Home/community-based/outpatient services Hospital/inpatient services Medication use Respite care	OOP: $898	NR	NR	Annual costs
Flanders (2007) [26]	USA	To compare direct costs of treatment of children with ASD, asthma, and diabetes	Secondary analysis of data (California Medicaid Medical database)	*N* = 731 (3–17 years)	Payer	Hospital/inpatient services Outpatient services Medication use	Insurer spending: $6436	NR	NR	Annual costs
Jarbrink (2007) [27]	UK	To show the major cost drivers among children with ASD	Survey (Respondents: parents)	*N* = 55 (< 18 years old)	Provider	Hospital/inpatient services Home/community-based/outpatient services Respite care Medication use Other	Provider spending: $3996	NR	NR	Annual costs
Leslie et al. (2007) [28]	USA	To examine the healthcare expenditures associated with ASDs in medical care settings	Secondary data analysis (Administrative claims database)	*N* = 613 (< 17 years old)	Payer Patient	Hospital/inpatient services Outpatient services Medication use	OOP + Insurer spending: $8531	NR	NR	Annual costs
Sharpe and Baker (2007) [29]	USA	To identify factors associated with financial problems in families that have a child with autism	Survey (Respondents: parents and care givers)	*N* = 333 (< 19 years old)	Patient	Hospital/inpatient services Outpatient services Respite care Medication use Other	NR	NR	NR	Annual costs
Shimabukuro et al. (2008) [30]	USA	To estimate medical expenditures for children with ASD who receive employer-based health insurance	Secondary analysis of data (employer-based health insurance claims: the MarketScan research databases)	*N* = 3481 (1–21 years)	Payer Patient	Hospital/inpatient services Home/community-based/outpatient services Respite care Medication use Other	OOP: $805 Insurer spending: $10,006	NR	NR	Annual costs
Knapp et al. (2009) [13]	UK	To estimate the costs of ASDs in the UK	Secondary analysis of data	Children with intellectual disability	Provider	Hospital/inpatient services Respite care Other	Provider spending: $11,946	NR	NR	Annual costs
				Children without intellectual disability			Provider spending: $30,936	NR	NR	
Young et al. (2009) [31]	USA	To examine the healthcare expenditures associated with ASDs in medical care settings.	Survey	*N* = 113 (2.5–21 years)	Patient	Hospital/inpatient services Outpatient services Respite care Medication use Other	OOP: $882	NR	NR	Annual costs
Wang et al. (2010) [32]	USA	To examine trends in health care expenditures associated with ASDs in state Medicaid programs	Secondary analysis of data (Medicaid data)	*N* = 69,542 (> 17 years old)	Payer	Hospital/inpatient services Home/community-based/outpatient services Respite care Medication use Other	Insurer spending: $33,360	NR	NR	Annual costs
Barrett et al. (2012) [33]	UK	To determine family out-of-pocket expenses and productivity losses, and explore the relationship between family characteristics and costs.	Randomized controlled trial	*N* = 152 (2–4 years)	Patient Provider	Hospital/inpatient services Home/community-based/outpatient services Respite care Medication use Other	OOP: $446 Provider spending: $2240	$539	$3792	6 months
Parish et al. (2012) [34]	USA	To investigate the association between state Medicaid spending for children with disabilities and the financial burden reported by families of children with autism.	Secondary analysis of data (National Survey of Children with Special Healthcare Needs)	*N* = 2082 (< 18 years old)	Patients	NR	OOP: $690	NR	NR	Annual costs
Peacock et al. (2012) [10]	USA	To compare medical expenditures between children with ASDs and without ASDs	Secondary analysis of data (Health insurance claims: MarketScan Medicaid Multi-State Databases)	*N* = 8398 (2–17 years)	Payer	Hospital/inpatient services outpatient services Medication use	Insurer spending: $14,777	NR	NR	Annual costs
Wang et al. (2012) [35]	China	To determine the health expenses incurred by families of children with ASD and those expenses in relation to total patient income and expenditures	Survey (Respondents: parents)	*N* = 290 (1–15 years)	Patient	Home/community-based/outpatient services Respite care Medication use Other	OOP: $2936	NR	NR	Annual costs
Al-Farsi et al. (2013) [36]	Oman	To measure medical expenditures in children with ASDs	Survey (Respondents: parents)	*N* = 150 (< 15 years old)	Patient	Home/community-based/outpatient services Respite care Medication use Other	OOP:$346	$961	NR	Monthly
Van Steensel et al. (2013) [37]	USA	To measure the societal costs of children with high-functioning ASD and comorbid anxiety disorder(s)	Survey (Respondents: parents)	*N* = 194 (7–18 years)	Patient	Home/community-based/outpatient services Respite care Medication use Other	OOP: $16,806	NR	NR	Annual costs
Raz et al. (2013) [38]	Israel	To measure the level of OOP expenditure for health services by families with autistic children	Survey (Respondents: parents)	*N* = 204 (4–10 years)	Patient	Home/community-based/outpatient services Respite care Medication use Other	OOP: $9930	NR	NR	Annual costs
Buescher et al. (2014) [9]	UK	To estimate lifetime societal economic costs among individuals with ASD	NR	NR	Patient	Medical services	Adults with ID: $8738 Adults without ID:$27,265	NR	NR	Annual costs
				NR	Patient	Medical services	Children with ID:$1578 Children without ID: $1030	NR	NR	Annual costs
	USA			NR	Patient	Medical services	Adults with ID: $32,630 Adults without ID:$16,316	NR	NR	Annual costs
				NR	Patient	Medical	Children with ID: $18,645 Children without ID: $9323	NR	NR	Annual costs
Horlin et al. (2014) [14]	Australia	To compare expenses between families whose children receiving immediate versus delayed diagnoses	Survey (Respondents: parents)	*N* = 521 (< 18 years old)	Patient	All healthcare	OOP: $6937	30,422 (loss of income of the parents and caregivers)	35,593	Annual costs
Lavelle et al. (2014) [12]	USA	To estimate the associations between ASD diagnoses and service use, caregiver time, and cost outcomes	Secondary analysis of data (National Health Interview Survey) and a study-specific survey	*N* = 258 (3–17 years old)	Patient Payer	Hospital/inpatient services Home/community-based/outpatient services Respite care Medication use Other	Insurer spending: $3618 OOP: $218	NR	NR	Annual costs
Thomas et al. (2014) [39]	USA	To investigate the association between school transition age and healthcare expenditures for children with ASD	Secondary analysis of data (Pooled data from the Medical Expenditure Panel Survey)	*N* = 337 (< 21 years old)	Patient	All healthcare	Median OOP: $490	NR	NR	Annual costs
Barrett et al. (2015) [40]	UK	To describe the services and associated costs for individual with ASD and	Cohort of adolescents with ASD and other special needs	*N* = 51(adolescents with autistic disorder) (9–14 years)	Provider	Hospital/inpatient services Home/community-based/outpatient services Respite care Medication use Other	Provider spending: $1231	NR	NR	6 months
				*N* = 45 (adolescents with other ASDs) (9–14 years)			Provider spending: $1999	NR	NR	
Byford et al. (2015) [41]	UK	To investigate the cost-effectiveness of a communication-focused therapy for pre-school children with ASD	Cohort study	*N* = 77 (PACT+ treatment as usual) (2–4 years)	Provider Patient	Hospital/inpatient services Home/community-based/outpatient services Respite care Medication use Other	OOP: $2896 Provider spending: $16,523	$1223	$20,645	13 months
				*N* = 75 (treatment as usual) (2–4 years)	Provider Patient		OOP: $1990 Provider spending: $5180	$842	$7923	
Parish et al. (2015) [42]	USA	To investigate the relationship between family financial burden and children’s health	Secondary analysis of data (Pooled 2000–2009 Medical Expenditure Panel Survey data)	*N* = 316 (< 18 years old)	Patient	NR	OOP: $904	NR	NR	Annual costs
Thomas et al. (2016) [43]	USA	To examine expenditures according to types of health insurance included private alone, Medicaid alone, and combined private and wrap-around Medicaid	Secondary analysis of data (Pooled data from the Medical Expenditure Panel Survey)	*N* = 346 (< 18 years old)	Patient	Hospital/inpatient services Home/community-based/outpatient services Respite care Medication use Other	OOP for Medicaid insured children: $156 OOP for private insured children: $1579 Medicaid spending: $8383 Private insurance spending: $3736	NR	NR	Annual costs
Barry et al. (2017) [44]	USA	To measure whether implementing autism mandates altered service use or spending among commercially insured children with ASD	Secondary analysis of data (Health insurance claim: Data from three national insurers)	*N* = 106,977 (< 21 years old)	Payer	Hospital/inpatient services Home/community-based/outpatient services Respite care Medication use Other	Mandate eligible: $767 Mandate ineligible: $600	NR	NR	Monthly
Chang et al. (2018) [45]	Taiwan	To compare the differences in dental utilization and expenditure between children and adolescents with and without ASD	Secondary analysis of data (Health insurance claim: The database of the National Health Research Institutes)	*N* = 1305 (< 18 years old)	Payer	Dental treatment	Insurer spending: $110	NR	NR	Annual costs
Vohra et al. (2017) [46]	USA	To investigate the prevalence and association of comorbidities with healthcare utilization and expenditures of fee-for service enrolled adults with and without ASD	Secondary analysis of data (Health insurance claim: Medicaid data)	*N* = 1772 (22–64 years)	Payer	Hospital/inpatient services Home/community-based/outpatient services Respite care Medication use Other	Insurer spending: $16,928	NR	NR	Annual costs
Candon et al. (2019) [47]	USA	To investigate whether mandates have heterogeneous effects on healthcare expenditure by insures and individuals with ASD	Secondary analysis of data (Health insurance claim)	N = 106,977 (< 21 years old)	Patient Payer	NR	OOP: $124 Insurer spending: $453	NR	NR	Monthly
Hong et al. (2019) [48]	South Korea	To estimate the economic burden of ASD in South Korea	Secondary analysis of data (Health insurance claim: The National Health Insurance Service)	*N* = 5653 (1–89 years)	Patient	Hospital/inpatient services Home/community-based/outpatient services	NR	NR	$2496	Annual costs
Li et al. (2019) [49]	Taiwan	To examine the cost and utilization of rehabilitation resources among children with ASD	Secondary analysis of data (Health insurance claim: The National Health Insurance Research Database)	*N* = 3227 (3–12 years)	Payer	Home/community-based/outpatient services	Insurer spending: $2100	NR	NNRM	Annual costs
Roddy and O’Neill (2019) [50]	Ireland	To measure the societal cost of childhood ASDs	Survey (Respondents: parents)	*N* = 222 (2–18 years)	Patient	Hospital/inpatient services Home/community-based/outpatient services Respite care Medication use Other	OOP: $2756	NR	NR	Annual costs
Zerbo et al. (2019) [11]	USA	To compare healthcare utilization patterns and cost among insured adults with ASD, adults with ADHD and adults with neither condition	Secondary analysis of data (Administrative claim data: The Kaiser Permanente Northern California (KPNC) database	*N* = 1507 (> 18 years)	Provider	Hospital/inpatient services Home/community-based/outpatient services Respite care Other	Provider spending: $7889	NR	NNRM	Annual costs
Zuvekas et al. (2021) [51]	USA	To estimate healthcare costs for US children with ASD	Secondary analysis of data (Two different surveys)	N = 45,944 (3–17 years)	Patient	Hospital/inpatient services Home/community-based/outpatient services Respite care Medication use Other	Medical Expenditure Panel Survey (MEPS: $4163 National Health Interview Survey (NHIS): $5955	NR	NR	Annual costs
Höfer et al. (2022) [52]	Germany	To estimate health service use and associated costs in children, adolescents and adults with ASD with and without intellectual disability	Survey (Respondents: parents)	*N* = 385 (4–67 years)	Patients	Hospital/inpatient services Home/community-based/outpatient services Respite care Other	$4208	NR	NR	Annual costs
Ames et al. (2021) [53]	USA	To calculate healthcare service utilization and cost among transition-age youth with ASD and other special healthcare needs	Secondary analysis of data (Administrative claim: Kaiser Permanente Northern California)	*N* = 4123 (14–25 years)	Provider	Hospital/inpatient services Home/community-based/outpatient services Respite care Medication use Other	Provider spending: $5302	NR	NR	Annual costs

All costs are in U.S. dollars and are adjusted to reflect 2021 USD

*ADHD* Attention-Deficit and Hyperactivity Disorder; *ASD* Autism spectrum disorder; *NR* Not reported; *UK* United Kingdom; *USA* United States of America; *OPP* out of pocket payment

### Direct healthcare costs

Included costs varied between studies, as illustrated in Table 3. Focusing on direct healthcare costs only, a number of studies reported that rehabilitation and psychological therapies constitute the greatest proportion of total healthcare-related expenditure for individuals with ASD. In the study by Wang et al., carried out in the USA, the highest percentage of healthcare expenditures was devoted to behavioral therapies (54.3%), followed by transportation and accommodation (18.4%), outpatient care (15.9%), and medication (8.2%) [54]. Similarly, Ganz et al., in the USA, estimated that behavioral therapies were the largest part of lifetime per capita direct medical expenditures (206,337 USD) of which was highest for children aged 3–7 years [55]. Mosadeghrad et al. also reported that rehabilitation services comprised the largest component (70%) of the annual ASD direct medical costs in their study conducted in Iran [56].

Outpatient and community-based services, such as physician appointments, were more commonly used than inpatient care and thus these services would impose a high economic burden on families of individuals with ASD in countries where such services are paid out of pocket. The study by Barrett et al. (2012), in the UK, reported a higher proportion of the included sample utilizing outpatient and community-based appointments, with 60, 57 and 48% of children with ASD having at least one contact with community pediatricians, general practitioners, and health visitors (nurses or midwives who have undertaken additional training in community public health nursing), respectively, over a 6 month period, compared to 10% of participants reporting an inpatient episode of care [33]. Similarly, the findings of Vohra et al., in the USA, indicated that following prescription drugs, outpatient visits were the second major contributor to healthcare expenditure [46].

The included studies suggest that prescription drugs make a major contribution to expenditure for households. Mosadghrad et al., in Iran, for example, estimated the average annual direct medical costs of ASD? were approximately 2215 USD of which 16% consisted of medication, compared to 7 and 6.5% for outpatient and inpatient visits, respectively [56]. Similarly, Flanders et al., in the USA, estimated the mean annual healthcare costs of prescription medication at 827 USD per person, compared to 222 USD per person for inpatient services [26], whilst Vorha et al. found prescription drugs to be the largest contributor to healthcare expenditure [56].

Eight studies (24.24% of included studies) reported that inpatient services, equipment and emergency room visits constituted a smaller proportion of total healthcare costs compared to outpatient services. For example, a study by Thomas et al. (2014), in the USA, showed that inpatient services constitute only 0.5% of expenditure compared to outpatient services (27.7%) [39]. Young et al. (2009), in the USA, reported that only 5 and 12.5% of individuals with ASD with public and private insurance coverage had used inpatient care but more than 70% of the participants had received rehabilitation services such as speech-language therapy and occupational therapy [31]. Of these, privately insured children unexpectedly had lower expenditure than those with public insurance. Additionally, in the study by Vohra and colleagues conducted in the USA, the mean annual cost of inpatient care constituted a lower proportion (10.99%) of total healthcare expenditure compared to outpatient visits (14.76%), prescription drug (20.47%), and emergency room visits (53.76%) [46].

### Indirect costs

Indirect costs were commonly estimated in studies involving children and young people and were focused on lost productivity for parents and carers. Three studies (9.09% of included studies) showed that indirect costs constitute a greater proportion of total costs (direct + indirect) compared to healthcare expenditures in families of individuals with ASD. Accordingly, a study by Al-Farsi et al., in Oman, indicated a higher monthly indirect cost (924 USD; 8.92% of total costs) in contrast to direct healthcare expenditure (328 USD; 2.99% of total costs) [36]. In their study, the mean loss of income due to lost employment opportunity in middle-high income families (978 USD) was greater than low-income families (254 USD). Similarly, in the study by Horlin et al., in Australia, loss of income of parents and caregivers made a substantially larger contribution to the total cost of ASD per person (30,000 USD; 47%) compared to healthcare expenditure (4800 USD; 13.67%) [14]. They also reported that loss of income for the first income quartile (19,500 USD) was lower than the third income quartile (48,700 USD).

In contrast, Mosadeghrad et al., carried out in Iran, reported mean annual indirect costs due to productivity loss of 1118 USD, which was a smaller contribution to total expenditure (16%) compared to direct medical costs (32%) and direct non-medical costs (52%) [19]. In the study by Hong et al., carried out in South Korea, indirect costs for individuals with ASD in the age group of 20–29 years were lower than direct costs per capita (863 USD; 40.12% of total costs), but were higher than direct costs in older age groups [48].

### Key determinants of healthcare costs among individuals with ASD

Table 4 summarises the major contributing factors to healthcare costs among individuals with ASD, and the direction of the relationship, as reported by included studies.

**Table 4:** Contributing factors to healthcare costs in individuals with ASD

Determinants of healthcare costs	Study
Severity of ASD (+)	(Järbrink and Knapp 2001), (Sharpe and Baker 2007), (Barrett, Byford et al. 2012), (Barrett, Byford et al. 2012), (Raz, Lerner-Geva et al. 2013), (Horlin, Falkmer et al. 2014), (Roddy and O’Neill 2019)
Associated disabilities (+)	(Järbrink and Knapp 2001), (Järbrink 2007), (Peacock, Amendah et al. 2012), (Lavelle, Weinstein et al. 2014), (Thomas, Parish et al. 2014), (Vohra, Madhavan et al. 2017), (Roddy and O’Neill 2019), (Page, McKenzie et al. 2021), (Buescher, Cidav et al. 2014)
Utilizing medical interventions (+)	(Sharpe and Baker 2007)
Age (+)	(Barrett, Byford et al. 2012), (Lavelle, Weinstein et al. 2014), (Thomas, Parish et al. 2014), (Parish, Thomas et al. 2015), (Chang, Wang et al. 2018), (Hong, Lee et al. 2020), (Buescher, Cidav et al. 2014)
Age (−)	(Barrett, Mosweu et al. 2015), (Barry, Epstein et al. 2017), (Li, Chen et al. 2019), (Page, McKenzie et al. 2021), (Research Ethics Committees Certificate 2019)
Being male (+)	(Hong, Lee et al. 2020),
Being female (+)	(Li, Chen et al. 2019), (Page, McKenzie et al. 2021), (Research Ethics Committees Certificate 2019)
Income (−)	(Sharpe and Baker 2007), (Thomas, Parish et al. 2014),
Income (+)	(Raz, Lerner-Geva et al. 2013), (Parish, Thomas et al. 2015), (Candon, Barry et al. 2019)
Health insurance (−)	(Parish, Thomas et al. 2012), (Thomas, Williams et al. 2016)
Rural residence (+)	(Wang, Zhou et al. 2012)
Rural residence (−)	(Parish, Thomas et al. 2015)
Parents’ education (+)	(Raz, Lerner-Geva et al. 2013), (Thomas, Parish et al. 2014), (Page, McKenzie et al. 2021)
Education level among adults with ASD (−)	(Page, McKenzie et al. 2021)
Living with parents (+)	(Raz, Lerner-Geva et al. 2013)
Living with a single mother head of household (−)	(Thomas, Parish et al. 2014)
Having at least one older sibling (−)	(Raz, Lerner-Geva et al. 2013)
Delay in diagnosis (+)	(Horlin, Falkmer et al. 2014),
Prescription medication (+)	(Lavelle, Weinstein et al. 2014), (Thomas, Parish et al. 2014)
Private insurance (+)	(Parish, Thomas et al. 2015),
Household size (−)	(Parish, Thomas et al. 2015)
Being visited in teaching hospital (+)	(Li, Chen et al. 2019)

### Associated morbidities

In some included studies, associated morbidities were one of the significant contributors to total healthcare expenditures in children with ASD. Van Steensel et al., in the USA, estimated the cost of children with ASD and comorbid anxiety disorder were four times higher than anxiety disorder alone, and 27 times higher than typically developing children [37]. Similarly, Peacock et al., in the USA, found that co-occurring conditions such as intellectual disability, Attention Deficit/Hyperactivity Disorder (ADHD) and seizure disorders can increase the overall costs in children with ASD [10]. For example, they report that average annual expenditures for children with ASD without associated conditions was only 7200 USD per person compared with 19,190 USD for children with ASD and an intellectual disability.

### Symptom severity

A number of included studies showed that increasing symptom severity was associated with increased costs. Raz et al., in Israel, suggests that individuals with a clinically more severe condition have a higher likelihood of very high costs (OR = 3.31; 95% CI 1.40–7.83) [38]. However, Van Steense et al., in the USA, found no significant relationship [37]. The findings of Horlin et al., in Australia, indicated that each additional symptom adds nearly 1400 USD per annum to the cost of ASD [14]. The authors also report that delay in diagnosis could lead to an increase in the number of ASD symptoms which in turn was associated with higher healthcare costs. Furthermore, a study by Barrett et al., in the UK, showed that fulfilling three functional domains concerning communication, social and repetitive impairments, cost an additional £125 per month, in comparison to children who met only two of the three criteria [33].

### Health insurance

Some included studies found that families in receipt of public health insurance, such as Medicaid, were more likely to demonstrate lower out of pocket costs compared with those in receipt of private insurance. A study by Parish et al., in the USA, showed that the median annual out of pocket costs in low-income families of Medicaid–insured individuals with ASD was 7 USD, compared with 160 USD for low-income families with private insurance coverage [42].

Studies also suggest that a lack of insurance coverage increases the probability of high out of pocket expenditures [1, 2]. Thomas et al., in the USA, found that out of pocket expenditures were significantly different by type of insurance such that children covered by private insurance had higher odds of high out of pocket spending compared with their counterparts covered by Medicaid [43]. In their study, the annual out of pocket per person expenditure for Medicaid insured children was estimated to be 150 USD compared to 1335 USD for those with private insurance coverage. In contrast, they found that the mean annual expenditures paid by Medicaid (per person) (7088 USD) was higher than private insurance companies (3151 USD). Similarly, Barry at al., in the USA, who investigated the role of commercial insurance in out of pocket expenditures, found commercial insurance to be associated with a 3.4% increase in out of pocket expenditure on health services and a 77 USD increase in monthly costs for ASD-specific services compared with public insurance [44].

### Age

Seven studies showed a positive association and three studies indicated a negative association between age and healthcare costs among individuals with ASD. In adults, Zerbo et al., in the USA, found that the annual heath care cost in adults aged 50 years and older (12,000 USD) was higher than for the 18–25 age group (5000 USD) [9].

In younger populations, some studies showed that younger age groups were associated with higher costs than older age groups. For example, Barrett et al. (2015), in the UK, found that for every one-year increase in age, total health, education and social care costs fell by £4917 among adolescents with autism and autism spectrum disorder aged between 14 and 17 [40]. Similarly, Shimabukuro et al., in the USA, reported that children with ASD in the age group 1–4 years had a higher mean annual cost (8040 USD) compared with older age groups [30]. In contrast, Barrett et al. (2012), in the UK, in another study focused on very young children with autism aged between 2 and 4 years, estimated that young children in higher ages (45–60 months) were likely to use more resources than those in lower ages (24–45 months) [33]. Their study showed that for every 1 month increase in age, healthcare costs increased by £7 per month.

### Socioeconomic status

Included studies suggest that households with higher socioeconomic status (SES) were more likely to pay out of pocket for healthcare in comparison to those with lower SES. For instance, Raz et al., in Israel, and Thomas et al., in the USA, reported that higher parental education and higher household income were associated with higher out of pocket expenditure in families of children with ASD [38, 39]. On the other hand, Parish et al., in the USA, cited that in middle and upper-income households, the financial strain was considerably higher for privately insured individuals compared to those with public insurance [42].

## Discussion

This systematic review aimed to identify factors contributing to healthcare costs among individuals with ASD. Our findings showed that therapy services, outpatient visits, and medications made the largest contributions to total direct healthcare costs of ASD compared to inpatient care in the included studies, suggesting ASD is primarily managed in the community. Studies show that individuals with ASD are more likely to use therapy services (such as behavioral, occupational and speech therapy) than those without ASD which in turn imposes high costs on families and health systems [10, 25].

Insurance coverage was an important factor influencing the use of services like psychological and rehabilitation therapies, which are known to be important for improving competencies and facilitating social and economic participation in individuals with intellectual disabilities [57]. For example, in countries like Iran, there is no insurance coverage for occupational and speech therapies and thus families of children with intellectual disabilities have to pay out of pocket, with rehabilitation services constituting over 70% of direct medical expenditure among children with ASD [58]. Similarly, behavioral therapies for individuals with ASD are not covered by the Chinese national health insurance program, which imposes a large economic burden on families [35]. There is, therefore, a need to consider public funding of such services in order to protect individuals with ASD and their families from catastrophic health expenditures in countries where such services are paid out of pocket [59, 60].

In countries with public insurance, such as the USA, included studies suggest that -children in receipt of public insurance had a lower out of pocket financial burden compared to uninsured and privately insured children [42]. Our review showed that public insurance can have a major role in protecting families, particularly those on low income, against high direct medical costs associated with ASD. It is important to note that some private insurance companies do cover the cost of therapeutic services for individual with ASD, but low-income families may be unable afford to purchase private coverage. In addition, private insurance companies tend to shift healthcare costs to patients through high copayments or deductibles which in turn increases out of pocket expenses for patients and their families [61] and increases the risk that families cannot afford to purchase insurance.

Included studies showed that outpatient visits and medications constituted a major share of direct medical costs among individuals with ASD [9, 25, 29]. Literature indicates individuals with ASD have higher rates of poorer physical and mental health conditions than their counterparts without ASD which require further healthcare utilization to address these problems [5]. The present review showed that associated comorbidities predict higher healthcare costs [10, 12, 27]. For example, Zebro et al. in the USA, showed that people with ASD had significantly higher utilization of outpatient visits for primary care (74.2% vs. 66.6%), mental health (43.3% vs. 33.2%), and laboratory services (60.9% vs. 54.4%) compared to peers with ADHD [11].

The impact of age on healthcare costs was conflicting, with some suggesting a positive association and others suggesting a negative association. For adults, older age was associated with higher costs. However, the opposite was true in some studies focused on children and young people, with evidence that younger ages are associated with higher physician visits, prescription medication and related costs among individuals with ASD [27, 38, 40, 44].

Whilst the focus of the current paper was on healthcare costs, some included studies took a broader perspective and investigated societal costs among individuals with ASD [27, 37, 50]. In these studies, education made a larger contribution to total annual costs compared to healthcare. For example, the findings of Jarbrink et al., in the UK, showed that the contribution of local government services, schooling and community support comprised 78% of the total cost, while the cost for healthcare was marginal and accounted for less than 5% of the total societal cost [27]. The findings indicate that severe learning disabilities not only are associated with higher healthcare costs, but also with higher education costs (e.g. specialized schooling, transportation etc.) for children with ASD that should be taken into consideration by policy makers.

A number of included studies estimated indirect costs, in addition to direct healthcare costs, and these studies suggest that indirect costs constitute a major part of the total cost of individuals with ASD [14, 24, 33]. In particular, productivity losses (reduced productivity whilst at work or time off work due to illness or caring responsibilities) were found to constitute a substantial proportion of total societal costs, with one study reporting a contribution of almost 10% for productivity losses compared to 7% for out of pocket expenses (7.36%) [33]. Similarly, Al–Farsi et al., in Oman, estimated monthly income loss due to lost employment opportunity at 830 USD, which was higher than monthly out-of-pocket expenses per child (295 USD) [36]. The findings suggest that families spend a considerable amount of time seeking treatment and providing informal (unpaid) care for their family members with ASD. Family plays a central role in the treatment plan of children with ASD through encouraging collaboration, sharing information, reducing emotional distress, empowerment, and joint decision making [62, 63]. Some studies show that accompaniment by family members or parents helps to reduce anxiety and provide comfort in children with ASD [64]. Consequently, households commonly shift a major part of their resources (e.g., time and money) towards providing needed interventions for their children with ASD which subsequently may have a detrimental impact on the family finances, as employment, and thus household income, is reduced whilst healthcare expenditures increases. This suggests that the wellbeing of families’ with children with ASD, including quality of life, employment status, income, education, and physical and mental health, needs to be addressed by policy makers and researchers, alongside the wellbeing of people with ASD.

## Limitations

Our study faced some limitations. Firstly, we were not able to compare total healthcare costs and out of pocket expenses between included studies because of heterogeneity in the cost components included in each study. Second, healthcare delivery and financing schemes varied across the included studies, making it difficult to compare healthcare costs between countries. Third, we did not include grey literature and non-English language articles which may have affected the findings and the generalizability of the present study. Finally, we only included studies that reported individual costs per person; aggregate medical and non-medical costs for individuals with ASD should be investigated at local and/or national levels in future studies.

## Conclusion

This systematic review showed that therapy services, outpatient visits and medications constituted a major share of total direct healthcare costs among individuals with ASD. Indirect costs were also considerable, being higher than direct healthcare costs in some studies. Key determinants of healthcare costs in the included studies were health insurance, associated morbidities, symptom severity, age and socioeconomic status. Our study supports the formulation of policy options to reduce financial risks in families of individuals with ASD.

## Data Availability

The dataset supporting the conclusions of this article is included within the article.

## Abbreviations

ASD: Autism spectrum disorder
SES: Socioeconomic status
UK: United Kingdom
USA: United States of America
USD: United States Dollar
OPP: Out of pocket payment
PICOS: Patient, Intervention, Comparator, Outcome, Study design
PRISMA: Preferred Reporting Items for Systematic Reviews and Meta-Analyses
NR: Not reported
